# ZmLSD1 Enhances Salt Tolerance by Regulating the Expression of *ZmWRKY29* in Maize

**DOI:** 10.3390/plants13202904

**Published:** 2024-10-17

**Authors:** Qiaolu Li, Rongrong Hu, Min Jiang, Wei Zhang, Xinyi Gao, Binglin Zhang, Weijuan Liu, Zhongyi Wu, Huawen Zou

**Affiliations:** 1 College of Agriculture, Yangtze University, Jingzhou 434025, China; 2023730076@yangtzeu.edu.cn (Q.L.); 18674002969@163.com (R.H.); zw220319@163.com (W.Z.); 202104518@yangtzeu.edu.cn (X.G.); zhangbl833@hotmail.com (B.Z.); 2 Tianmen Academy of Agricultural Sciences, Tianmen 431700, China; JM900213@163.com; 3 Institute of Biotechnology, Beijing Academy of Agriculture and Forestry Sciences, Beijing Key Laboratory of Agricultural Gene Resources and Biotechnology, Beijing 100097, China

**Keywords:** ZmLSD1, transcription factor, maize, salt stress

## Abstract

Salt stress significantly impairs plant growth, presenting a challenge to agricultural productivity. Exploring the regulatory mechanisms underlying salt stress responses is critically important. Here, we identified a significant role for the maize LESION-SIMULATING DISEASE transcription factor, ZmLSD1, in enhancing salt stress response. Subcellular localization analysis indicated that ZmLSD1-GFP was localized in the nucleus in the maize protoplast. Overexpressing *ZmLSD1* in maize obviously enhanced the tolerance of plants to salt stress. Physiological analysis indicated that overexpressed *ZmLSD1* in maize could mitigate the accumulation of H_2_O_2_ and MDA content exposed to salt stress. RNA-seq and qPCR-PCR analyses showed that ZmLSD1 positively regulated *ZmWRKY29* expression. ChIP-qPCR and EMSA experiments demonstrated that ZmLSD1 could directly bind to the promoter of *ZmWRKY29* through the GTAC motif both in vitro and in vivo. Overall, our findings suggest that ZmLSD1 plays a positive role in enhancing the tolerance of maize to salt by affecting *ZmWRKY29* expression.

## 1. Introduction

Rising global soil salinity levels lead to the increase in salt ions, such as sodium and chloride, which impair plant water uptake, disrupt metabolic processes including photosynthesis, and diminish growth and productivity [1,2]. Plants have evolved a range of adaptive mechanisms to mitigate salt stress. Generally, plants sense stimulus signals from the cell surface to the interior, thereby regulating gene responses to salt stress at both the physiological and biochemical levels. Numerous transcription factors, including bZIP, MYB, NAC, WRKY, and ZFPs, play crucial roles in the salt stress response [3,4,5,6,7]. 

Zinc finger proteins (ZFPs) are a widely prevalent family of transcription factors throughout the plant kingdom [8]. These proteins are distinguished by unique motifs composed of cysteine and histidine residues, which form structures akin to fingers capable of binding zinc ions [9]. Given the amino acid composition involved in zinc binding, ZFPs are categorized into distinct classes, including C2H2, C2HC, C2HC5, etc. [9]. In higher plants, a growing amount of evidence reveals the functions of ZFPs in various physiological developmental processes [10,11]. For instance, C2H2 zinc finger proteins, including AtZFP5 and AtZFP6, regulate trichome initiation of inflorescence organs in *Arabidopsis* through integrating gibberellin and cytokinin signaling [12,13]. In cucumber, a C2H2 zinc-finger transcription factor, CsSBS1, plays a crucial role in affecting spine base formation and size [14]. Furthermore, ZFPs play a crucial role in enhancing plant tolerance to abiotic stresses [15]. For instance, OsDRZ1 enhances the tolerance of rice to drought stress by reducing the accumulation of reactive oxygen species (ROS), increasing the proline level, and inducing the antioxidant system [16]. In wheat, overexpression of *TaZFP1B* improves drought tolerance during key developmental stages [17]. In tomato, SlZF3, a zinc finger protein, positively regulates ascorbic acid biosynthesis, thereby improving the plant salt stress tolerance [18]. The maize genome encodes ZFPs. Among them, several ZFPs have been identified, such as ZmC2H2-149, *ZmZF1*, *ZmAN13*, and *ZmELF6* [10,15,19]. Given maize’s rich diversity of ZFPs, ongoing research is essential to fully understand their functional mechanisms during salt stress. 

The LESION-SIMULATING DISEASE 1 (LSD1) transcription factor, belonging to the C2C2 zinc finger family, is a key factor in regulating cell death in *Arabidopsis thaliana* [20]. Since its identification, extensive functional research has underscored the importance of the *LSD1* gene. Mutants of *Arabidopsis AtLSD1* exhibit pronounced cell death phenotypes exposed to cold stress [21]. The rice ortholog OsLOL2, an ortholog of AtLSD1, contributes to both plant growth and resistance to bacterial blight [22]. In wheat, TaLSD1 acts as a negative regulator of hypersensitive cell death, thereby conferring protection against stripe rust infection [23]. However, the specific function of ZmLSD1 in regulating salt response remains to be elucidated.

Maize is one of the world’s most important cereal crops, serving as a staple food for millions of people globally. However, soil salinity is a significant constraint on maize production in many regions, reducing yields and threatening food security [24]. Thus, understanding the molecular mechanisms of salt tolerance is crucial. This research highlights the role of ZmLSD1 as a key factor in regulating the tolerance of maize to salt stress. It reveals that ZmLSD1 enhances salt tolerance by modulating the expression of the downstream gene *ZmWRKY29*. These findings elucidate ZmLSD1’s regulatory role in salt stress response, providing critical insights for improving maize’s genetic resistance to salt.

## 2. Results

### 2.1. ZmLSD1 Enhances the Tolerance of Maize to Salt Stress

**Figure 1:**
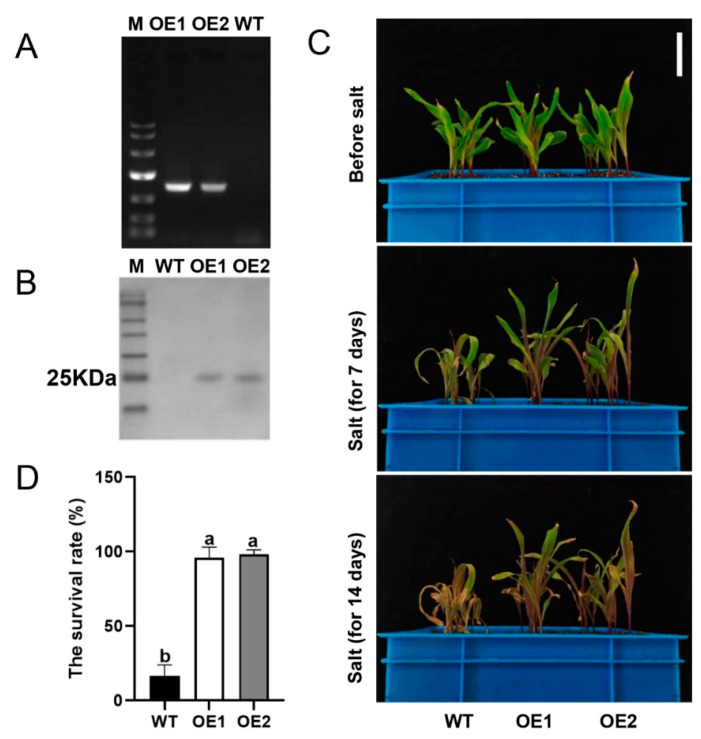
Overexpressing *ZmLSD1* in maize improves plant survival rates under salt stress. (**A**) PCR detection of transgenic maize lines. (**B**) Protein level of ZmLSD1 in transgenic maize lines. (**C**) Phenotype of wild type and *ZmLSD1* overexpressors under salt stress. Ten-day-old seedlings were treated with 200 mmol/L NaCl for seven days and fourteen days. Scale bar: 5 cm. (**D**) Survival rate of wild type and *ZmLSD1* overexpressors after 14 days of treatment with 200 mmol/L NaCl in (**C**). The experiment shown in (**C**,**D**) was performed at least three times. Error bars in (**D**) indicate SD (*n* = 12). Different letters indicate a significant difference (*p* < 0.05).

To investigate the role of ZmLSD1 in maize under salt stress, we created a transgene containing the *ZmLSD1* coding sequence driven by the *35S* promoter and stably introduced it into the maize inbred line KN5585. As shown in Figure 1A, no amplified strips were detected in the leaves of wild-type (WT) plants but were present in two independent *ZmLSD1*-overexpressing lines (OE1 and OE2) via agarose gel electrophoresis experiments. The Western blotting result was consistent with the PCR findings (Figure 1B), confirming that these two independent lines could be used for conducting salt hypersensitivity assays. 

Then, 10-day-old seedlings were subjected to treatment with NaCl (200 mmol/L) for 7 days. No noticeable differences in the growth and appearance of maize seedlings between the wild type and *ZmLSD1* overexpressors were observed under normal conditions. However, the wild-type maize seedlings exhibited wilting symptoms earlier than the *ZmLSD1* overexpressors after being exposed to 200 mmol/L NaCl (Figure 1C). The *ZmLSD1* overexpressors exhibited a nearly 100% survival rate, while only 17% of the WT plants survived (Figure 1C,D). These results indicate that overexpressing *ZmLSD1* obviously enhances the tolerance of maize to salt stress.

### 2.2. ZmLSD1 Mitigates Salt-Induced Oxidative Damage in Maize

**Figure 2:**
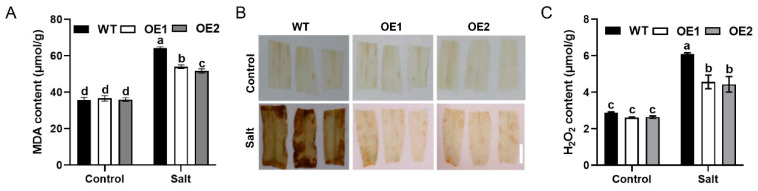
Effect of ZmLSD1 on lipid peroxidation and H_2_O_2_ accumulation under salt stress. (**A**) MDA content. (**B**) Detection of H_2_O_2_ accumulation using DAB staining. Scale bar: 1 cm. (**C**) H_2_O_2_ content. The experiments were performed three times. Error bars in (**A**,**C**) indicate SD (*n* = 3). Different letters indicate a significant difference (*p* < 0.05).

In plants, salt stress commonly induces oxidative damage. Thus, the content of MDA was measured. Under NaCl treatment, the MDA content in the *ZmLSD1* overexpressors was lower than that in the wild type (Figure 2A). To assess the effect of ZmLSD1 on H_2_O_2_ (a contributor to oxidative damage) levels under salt stress conditions, DAB staining was first used. The DAB experiments revealed that the leaves of the *ZmLSD1* overexpressors, indicated by shallow brown precipitation, had a lower H_2_O_2_ level compared with the wild type under salt stress treatment conditions (Figure 2B). Next, we assessed the content of H_2_O_2_ in the *ZmLSD1* overexpressor plants and the wild-type plants exposed to salt stress. As shown in Figure 2C, there were no significant differences observed between the groups without salt stress treatment. However, in the presence of NaCl, the *ZmLSD1* overexpressors displayed lower content of H_2_O_2_ compared to the wild-type plants. Hence, overexpression of *ZmLSD1* alleviates oxidative damage to enhance salt tolerance in maize.

### 2.3. ZmLSD1 Is Located in the Nucleus

**Figure 3:**
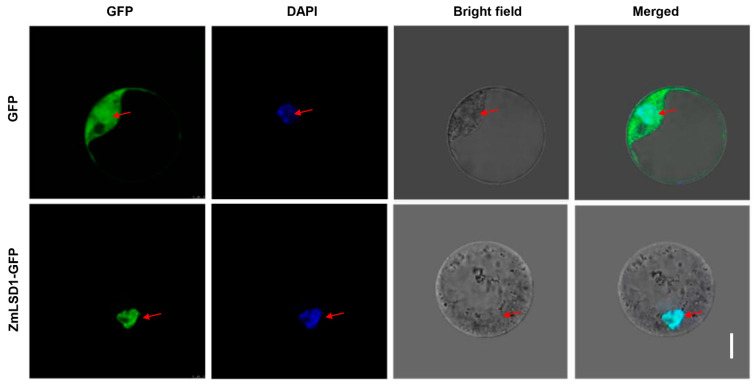
Subcellular localization of ZmLSD1. ZmLSD1-GFP constructs were introduced into maize protoplasts for transient expression. Nuclei (red arrow) were stained with DAPI (in blue). Scale bar: 5 μm.

To verify the subcellular localization of ZmLSD1, ZmLSD1-GFP fusion was transiently expressed in maize leaf protoplasts. Remarkably, a strong GFP signal was exclusively detected within the nuclei, coinciding with the blue fluorescence from diamino phenylindole (DAPI) staining, serving as a marker for the nucleus. Meanwhile, the GFP signals in leaves that only transiently overexpressed *GFP* alone were observed in the plasma membranes, cytoplasm, and nuclei (Figure 3). These results strongly indicate that ZmLSD1 is located in the nucleus.

### 2.4. ZmLSD1 Positively Regulates ZmWRKY29 Expression Exposed to Salt Stress

**Figure 4:**
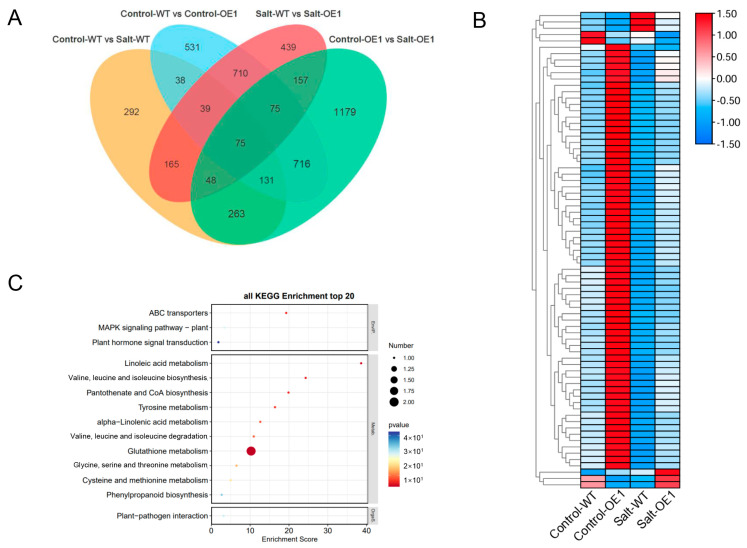
Transcriptome analysis identifies key genes regulated by ZmLSD1 under salt stress treatment. (**A**) Venn diagrams analyzing number of DEGs in wild type and *ZmLSD1* overexpressors treated with or without 200 mmol/L NaCl. (**B**) Heatmap analysis of 75 DEGs overlapping/shared in (**A**). (**C**) KEGG pathway analysis of genes in (**B**).

To uncover the molecular mechanism underlying the role of ZmLSD1 in salt stress response, we conducted transcriptome analyses on *ZmLSD1*-overexpressing plants and WT plants, both subjected to treatments with and without 200 mmol/L NaCl. As depicted in Figure 4A, the Venn diagrams illustrated the comparative analysis of DEGs. The analysis revealed that 1051 DEGs in the WT plants were specifically expressed between the control and salt conditions, while 2644 DEGs were found in the group of overexpressing plants. After salt treatment, 1708 DEGs were uniquely identified in the WT compared to the overexpressing plants. A total of 2315 DEGs were specifically expressed in the *ZmLSD1* overexpressor and WT plants under normal conditions. Additionally, the Venn diagram displays some of the 75 DEGs overlapping/shared between the two maize materials in response to salt stress (Figure 4A). The heatmap depicted 75 DEGs (Table S3) with unique expression profiles in the *ZmLSD1* overexpressor under salt treatment conditions compared to the WT (Figure 4B). To identify the biological processes associated with ZmLSD1, we performed KEGG enrichment analysis on the DEGs shared among the previously mentioned comparisons. The pathways related to ABC transports, glutathione metabolism, and metabolics were especially enriched (Figure 4C). 

**Figure 5:**
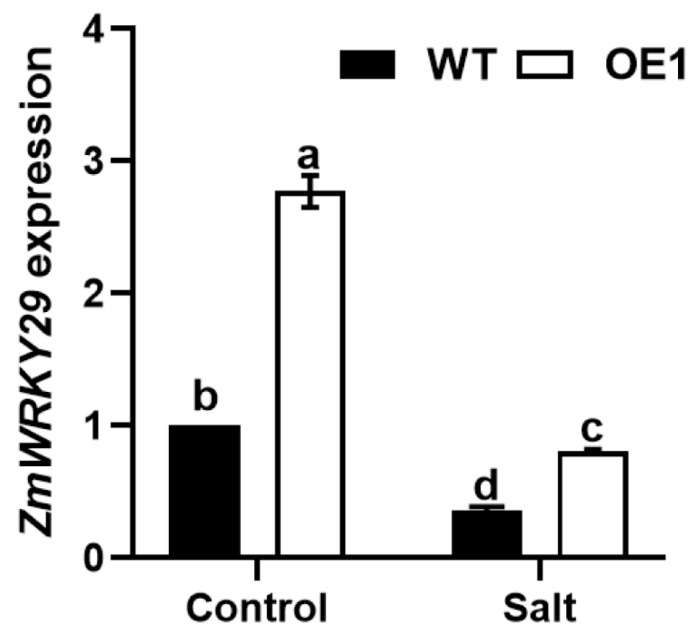
The expression level of *ZmWRKY29* in the *ZmLSD1* overexpressor and WT under salt stress. Ten-day-old seedlings were subjected to a 48-h treatment with 200 mmol/L NaCl. Subsequently, the second leaves were collected from the seedlings for qRT-PCR analysis. Error bars indicate SD (*n* = 3). Different letters indicate a significant difference (*p* < 0.05).

Given that WRKY transcription factors play a significant role in plant responses to salt stress [25,26], we validated RNA-seq data accuracy through the qRT-PCR analysis of one potential *WRKY* candidate gene, *ZmWRKY29* (Zm00001d043569). As shown in Figure 5, *ZmWRKY29* expression in the *ZmLSD1* overexpressor was much higher compared to that in the WT plant under NaCl treatment conditions. Consequently, these findings indicate that ZmLSD1 plays a positive regulatory role in *ZmWRKY29* expression exposed to salt stress.

### 2.5. ZmLSD1 Directly Binds to ZmWRKY29 Promoter In Vitro and In Vivo

**Figure 6:**
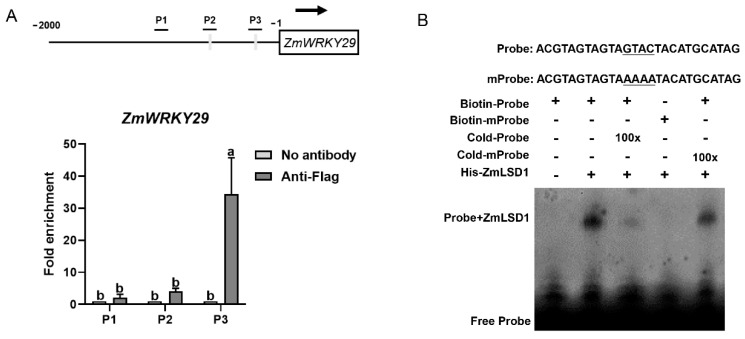
ZmLSD1 directly binds to *ZmWRKY29* promoters. (A) ChIP-qPCR verified that ZmLSD1 directly bound to the *ZmWRKY29* promoter in *vivo*. P2 (−600 to −597 bp) and P3 (−232 to −229 bp) contained the GTAC motif, but P1 did not have the GTAC motif. Chromatin was extracted from the *ZmLSD1* overexpressor and subjected to immunoprecipitation using an anti-Flag antibody. qPCR was used to assess expression levels with a reference value of 1 assigned to the control (without antibody). (**B**) EMSA showed that ZmLSD1 bound to the *ZmWRKY29* promoter in *vitro*. The His-ZmLSD1 purified protein was incubated with a biotin-labeled probe (referred to as Biotin-Probe), while a 100-fold excess of an unlabeled probe (referred to as Cold-Probe) was used as a competitive control. Error bars in (**A**) indicate SD (*n* = 3). Different letters indicate a significant difference (*p* < 0.05).

Considering that ZmLSD1 exhibited a positive regulatory effect on *ZmWRKY29* expression under the NaCl treatment (Figure 5), we aimed to explore whether ZmLSD1 binds directly to the promoter of *ZmWRKY29*. First, ChIP-qPCR was performed using the *ZmLSD1-Flag* overexpressor plant (OE1). Two binding sites, P2 (−600 to −597 bp) and P3 (−232 to −229 bp), containing the GTAC sequence, as well as a non-binding region, P1, without GTAC, were identified in the *ZmWRKY29* promoter. As shown in Figure 6A, ZmLSD1 displayed strong binding to the P3 region but did not show significant binding to the P1 or P2 regions of the promoter (Figure 6A).

To further confirm the interaction between ZmLSD1 and the *ZmWRKY29* promoter, EMSA was conducted. The P3 region containing the GTAC sequence (−243 to −218 bp) was synthesized and labeled as Biotin-Probe. A 100-fold excess of an unlabeled probe was used as competitors. As shown in Figure 6B, ZmLSD1 exhibited no binding affinity to the biotin-labeled mutant probe but showed a direct binding affinity to the biotin-labeled probes. A 100-fold excess of unlabeled competitive probes significantly disrupted this interaction, while the disruption was restored by adding a 100-fold excess of unlabeled competitor mutant probes (Figure 6B). These results suggest that ZmLSD1 directly binds to the *ZmWRKY29* promoter in *vitro* and in *vivo*.

## 3. Materials and Methods

### 3.1. Plant Materials and Growth Conditions

*Zea mays* L. KN5585 inbred line (provided by the laboratory of maize molecular biology under abiotic stress, Yangtze University) is a frequently used line in China for maize genetic transformation. Plants were planted in pots containing a soil mixture, then cultivated in a greenhouse with a 14-h period of light at 28 °C followed by a 10-h period of darkness at 22 °C. 

### 3.2. Generation of Transgenic Plants

The *ZmLSD1* CDS was inserted into the NEWMOL vector. The recombinant vector, NEWMOL-*ZmLSD1*, was introduced into *Agrobacterium* EHA105. Then, *Agrobacterium* EHA105 carrying *ZmLSD1* was transformed into KN5585 [27]. Positive transformants were identified through PCR analysis using specific primers (Table S2). Then, the protein level of ZmLSD1 in plants was assessed using Western blotting [28]. Briefly, maize leaf proteins were extracted using a plant total protein extraction kit (Sangon Biotech, Shanghai, China). The proteins were separated by 12% SDS-PAGE and transferred to a PVDF membrane. After blocking with 5% skim milk powder, the membrane was incubated with primary antibodies overnight at 4 °C, followed by incubation with secondary antibodies for 2 h at room temperature. Signals were detected using a Tanon 5200 Multi camera (Tanon, Shanghai, China). 

### 3.3. Phenotype Analysis

For salt stress treatment, ten-day-old seedlings of *ZmLSD1* overexpressor and wild-type (WT) seedlings were exposed to a NaCl (200 mmol/L) solution for an additional fourteen days. Subsequently, the survival rate of seedlings was recorded. The seedlings were photographed on days 7 and 14 following the salt treatment. Additionally, plants grown in pot soil were irrigated weekly with either water or a 200 mmol/L NaCl solution.

### 3.4. Subcellular Localization of ZmLSD1

The *ZmLSD1* CDS was inserted into the PYBA1132 vector, generating the plasmid PYBA1132-*ZmLSD1*-*GFP*. Protoplasts derived from maize seedling leaves were isolated [29]. One milliliter of maize protoplasts (typically 5 × 10^5^ cells per milliliter) was transfected with 100 μg of PYBA1132-*ZmLSD1*-*GFP*, employing the PEG–calcium-mediated method. Subsequently, the protoplasts were cultured in a dark environment for 16 h at 25 °C. Then, the fluorescence signal was observed.

### 3.5. Physiological Index Analysis

After treatment with NaCl (200 mmol/L) for seven days, the second leaf was flash-frozen in liquid nitrogen until future utilization. The content of malondialdehyde (MDA) was assessed as described by Yan et al. [30]. For DAB staining, the leaf tissues were exposed to a solution containing 1 mg/mL of 3,3′-diaminobenzidine (DAB). After incubating and washing, photographs were taken. H_2_O_2_ levels were also quantified using a H_2_O_2_ assay kit (Solarbio, Beijing, China). Fresh maize leaves were homogenized in cold acetone and centrifuged to obtain the supernatant. Titanium sulfate and ammonium hydroxide were added to the supernatant to form a precipitate, which was subsequently centrifuged again. The resulting pellet was dissolved in sulfuric acid, and the H_2_O_2_ concentration was measured at 415 nm.

### 3.6. Transcriptome Analysis

Ten-day-old seedlings of the wild type and *ZmLSD1* overexpressors were treated with or without NaCl (200 mmol/L) for 48 h, then the second leaf was flash-frozen in liquid nitrogen for RNA-Seq analysis. Total RNA was extracted with an RNA Extraction Kit (ABclonal, Wuhan, China). The RNA-seq data were normalized using FPKM values. Significant differentially expressed genes (DEGs) were determined by assessing expression levels with an absolute fold change ≥ 2. The Venn diagram and heatmap were constructed using TBtools software (v2.034). DEG enrichment analysis was employed using OEIOTECH software (https://cloud.oebiotech.com/, accessed on 14 October 2024).

### 3.7. qRT-PCR Analysis

To verify the accuracy of the transcriptome sequencing results, the relative expression levels of *ZmWRKY29* were analyzed in leaves from both wild-type and *ZmLSD1* overexpressor plants under normal and salt stress conditions. Quantitative real-time PCR (RT-qPCR) analysis was conducted with a CFX96 Touch system using an SYBR qPCR Master Mix kit (Vazyme, Nanjing, China). Relative expression levels were determined using the 2^−ΔΔCT^ method, referencing *ZmActin2* as a control (Table S2). Each analysis included three biological replicates and three technical replicates.

### 3.8. ChIP-qPCR

The ChIP-qPCR experiment was performed as described previously [30]. Maize leaves were fixed through vacuum infiltration with 1% formaldehyde, and cross-linking ceased with the addition of 0.125 M glycine. Subsequently, chromatin was isolated, fragmented via sonication, and immunoprecipitated utilizing an anti-Flag antibody (Solarbio, Beijing, China). Enriched DNA fragments were quantified via qPCR with specific primers (Table S2).

### 3.9. EMSA Test

The EMSA experiment was performed as previously described [30]. The *ZmLSD1* CDS was amplified and cloned into the vector pET-30a. The recombinant plasmid, pET-30a-*ZmLSD1*, was introduced into *Escherichia coli* BL21 (DE3) cells and inducted with 0.5 mM IPTG for 6 h at 30 °C. Subsequently, the purified His-ZmLSD1 proteins were incubated with biotin-labeled *ZmWRKY29* promoter fragments according to the Chemiluminescence EMSA kit protocol (Beyotime, Shanghai, China). An excess of 100× cold probe or mutated cold probe was used to compete with the biotin-labeled probe. Chemifluorescent signals were detected using a Tanon 5200 Multi camera. 

## 4. Discussion

Zinc finger protein transcription factors are critical regulators of both plant growth and development, particularly under stress conditions [31]. LSD1, a member of the C2C2 zinc finger protein family [32], has been shown through functional analyses to play a central role in modulating cell death, disease resistance, and responses to abiotic stress [13,17]. In Arabidopsis, AtLSD1 regulates cell death via its influence on *AtEDS1* and *AtPAD4*, two key components of disease resistance signaling [33]. Additionally, AtLSD1 is implicated in the regulation of lysogenic aerenchyma formation under hypoxic stress [34]. In *Glycine max*, several *GmLSD* genes are induced by drought stress [35], suggesting that LSD genes contribute to abiotic stress responses, although their precise mechanisms remain unclear. Here, our findings established ZmLSD1 as a positive regulator influencing maize response to salt stress based on the following results: First, overexpression of *ZmLSD1* improved the plant survival rates under salt stress (Figure 1). Secondly, the MDA content and H_2_O_2_ level in *ZmLSD1* overexpressors caused by salt stress were much lower compared to those in the wild type (Figure 2). 

Plants must enhance their mechanisms for managing oxidative damage to better withstand elevated abiotic stress. For instance, *SlNPR1* regulates tomato plants’ cold stress resistance by reducing MDA and H_2_O_2_ levels, thereby alleviating oxidative damage [36]. In tobacco, overexpression of tomato *SlTpx* enhances the plant’s tolerance to salt stress by promoting the scavenging of H_2_O_2_ [37]. Our results also revealed that the *ZmLSD1*-overexpressing plants exhibited reduced levels of MDA and H_2_O_2_ compared to the wild-type plants under salt stress (Figure 2). These findings uncover a novel regulatory pathway in which ZmLSD1 may enhance the regulation of antioxidant defense systems, leading to reduced levels of MDA and H_2_O_2_. This process alleviates oxidative damage and improves the survival rate of maize plants under salt stress.

Transcription factors are typically localized within the nucleus, where they play critical roles in regulating gene expression. Consistent with previous studies [7], our results confirm the nuclear localization of ZmLSD1 (Figure 3), further supporting its involvement in nuclear gene regulatory processes. LSDs could modulate the expression of downstream genes to regulate the stress response [38]. For instance, mutation of the *AtLSD1* gene in *Arabidopsis* causes changes in more than 2100 genes [38]. In this study, transcriptome sequencing revealed 75 DEGs shared between the wild-type and overexpression transgenic lines exposed to salt stress, potentially regulated by ZmLSD1 (Figure 4). Notably, our findings indicated that ZmLSD1 influenced the expression of a defense-related gene, namely *ZmWRKY29* [39]. Chromatin immunoprecipitation (ChIP) assays have further isolated 27 genes that are differentially enriched in both *lsd1* mutants and *LSD1*-overexpressing *Arabidopsis* plants [40]. This indicates that LSD1 could act as a transcriptional regulator in *Arabidopsis*. Here, we identified that ZmLSD1 can directly bind to the GTAC elements in the *ZmWRKY29* promoter both in *vitro* and in *vivo*, as demonstrated by ChIP-qPCR and EMSA experiments. WRKY transcription factors are a critical group extensively involved in maize’s response to salt stress. For instance, ZmWRKY86 has been shown to directly interact with the promoters of salt stress-associated genes, including Zm00001d020840 and Zm00001d046813, thereby regulating salt stress tolerance [41]. ZmWRKY114 acts as a negative factor of salt stress responses through an ABA-dependent pathway [42]. Additionally, a study indicated that ZmWRKY104 enhanced salt tolerance by upregulating *ZmSOD4* expression in maize [30]. In the present study, the expression of *ZmWRKY29* was markedly suppressed upon exposure to salt stress; however, this suppression is attenuated in *ZmLSD1*-overexpressing lines (Figure 5). 

**Figure 7:**
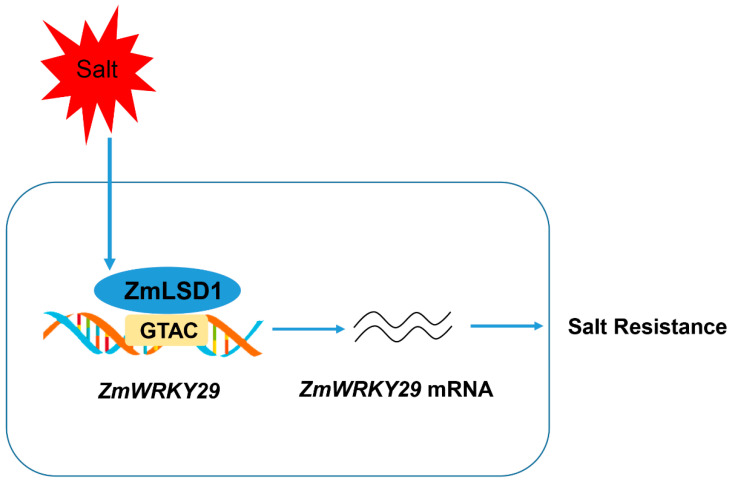
A model of ZmLSD1 activity in maize salt response. ZmLSD1 plays a positive role in enhancing the salt tolerance of maize by binding to the *ZmWRKY29* promoter and affecting its expression.

Taken together, our study offers valuable insights into the complex regulation of the salt stress response in maize, involving ZmLSD1 and ZmWRKY29, as illustrated in the proposed model (Figure 7). ZmLSD1 plays a positive role in enhancing the salt tolerance of maize by binding to the *ZmWRKY29* promoter and affecting its expression. Wider adoption of *ZmLSD1* overexpressors in maize cultivation could augment salt stress tolerance, potentially resulting in heightened yields amidst challenging environmental conditions.

## Data Availability

Data available upon request.

## Supplementary Materials

The following supporting information can be downloaded at https://www.mdpi.com/article/10.3390/plants13202904/s1. Table S1. The detected genes with FPKM in maize. Table S2. Primers used in this study. Table S3. The 75 DEGs in the heatmap.
